# GATA3-Driven Th2 Responses Inhibit TGF-β1–Induced FOXP3 Expression and the Formation of Regulatory T Cells

**DOI:** 10.1371/journal.pbio.0050329

**Published:** 2007-12-27

**Authors:** Pierre-Yves Mantel, Harmjan Kuipers, Onur Boyman, Claudio Rhyner, Nadia Ouaked, Beate Rückert, Christian Karagiannidis, Bart N Lambrecht, Rudolf W Hendriks, Reto Crameri, Cezmi A Akdis, Kurt Blaser, Carsten B Schmidt-Weber

**Affiliations:** 1 Swiss Institute of Allergy and Asthma Research Davos (SIAF), Davos-Platz, Switzerland; 2 Department of Pulmonary Medicine, Erasmus Medical College, Rotterdam, The Netherlands; 3 Division of Immunology and Allergy, University Hospital of Lausanne (CHUV), Lausanne, Switzerland; 4 Department of Immunology, Erasmus Medical College, Rotterdam, The Netherlands; 5 Department of Allergy and Clinical Immunology, Imperial College London, London, United Kingdom; National Jewish Medical and Research Center, United States of America

## Abstract

Transcription factors act in concert to induce lineage commitment towards Th1, Th2, or T regulatory (T_reg_) cells, and their counter-regulatory mechanisms were shown to be critical for polarization between Th1 and Th2 phenotypes. FOXP3 is an essential transcription factor for natural, thymus-derived (nT_reg_) and inducible T_reg_ (iT_reg_) commitment; however, the mechanisms regulating its expression are as yet unknown. We describe a mechanism controlling iT_reg_ polarization, which is overruled by the Th2 differentiation pathway. We demonstrated that interleukin 4 (IL-4) present at the time of T cell priming inhibits FOXP3. This inhibitory mechanism was also confirmed in Th2 cells and in T cells of transgenic mice overexpressing GATA-3 in T cells, which are shown to be deficient in transforming growth factor (TGF)-β–mediated FOXP3 induction. This inhibition is mediated by direct binding of GATA3 to the FOXP3 promoter, which represses its transactivation process. Therefore, this study provides a new understanding of tolerance development, controlled by a type 2 immune response. IL-4 treatment in mice reduces iT_reg_ cell frequency, highlighting that therapeutic approaches that target IL-4 or GATA3 might provide new preventive strategies facilitating tolerance induction particularly in Th2-mediated diseases, such as allergy.

## Author Summary

Specific immune responses against foreign or autologous antigens are driven by specialized epitope-specific T cells, whose numbers expand upon recognition of antigen found on professional antigen-presenting cells. The subsequent maturation process involves the differentiation of certain T cell phenotypes such as pro-inflammatory cells (Th1, Th2, Th17) or regulatory T (T_reg_) cells, which serve to keep the immune response in check. The current study focuses on the role of two key transcription factors—FOXP3 and GATA3—in controlling the commitment of these cells. We demonstrate that the Th2 cytokine IL-4 inhibits the induction of FOXP3 and thus inhibits the generation of inducible T_reg_ cells. We show that IL-4–induced GATA3 mediates FOXP3 inhibition by directly binding to a GATA element in the FOXP3 promoter. We hypothesize that therapeutic agents aimed at neutralizing IL-4 could be a novel strategy to facilitate inducible T_reg_ cell generation and thus promotion of tolerance in allergies and other Th2-dominated diseases.

## Abstract 3

It is shown that Th2 responses prevent the generation of inducible T_reg_s. This is mediated by IL-4 induction of GATA3, which binds directly to and represses the FOXP3 promoter. This mechanism is likely to be relevant in the induction of immunotolerance, particularly in allergic diseases.

## Introduction

Effective immune responses are characterized by T cell activation, which directs adaptive and innate immune responses to kill pathogens efficiently. Dependent on the pathogen, T cells differentiate into different subtypes, such as Th1 or Th2 cells, which are most efficient in defeating microbial or parasitic invaders respectively. A hallmark of Th1 and Th2 differentiation pathways is the exclusiveness of the individual phenotype leading to either Th1 or Th2, but not to mixed populations. The exclusiveness of this mechanism is provided by a polarization process, where Th2 differentiation inhibits Th1 commitment and vice versa. Specifically interleukin 4 (IL-4)–induced STAT6 and GATA3 inhibit differentiation into Th1 cells in the early phase of commitment [1,2]. GATA3 is sufficient to induce a Th2 phenotype [3] and acts not only through the induction of IL-4, IL-5 and IL-13, the Th2 cytokines, but also through the inhibition of Th1 cell-specific factors [3]. Recently, it was shown that T-bet directly modulates GATA3 function, suggesting that transcription factors compete in the early differentiation phase of T cells, potentially integrating environmental signals to finally imprint the T cell phenotype [4,5]. A GATA3-dominated immune response has been shown to be essential in airway hyperresonsiveness [6] and IL-4–dominated responses can break antigen-specific immune tolerance [7]. Overexpression of a dominant negative form of GATA3 [8] or treatment with antisense-mediated GATA3 blockade [9] decreased the severity of the allergic airway hyper-responsiveness.

The discovery of regulatory T (T_reg_) cells highlights another phenotype of T cells, which is essential for tolerance against self-antigens. Naturally occurring, thymus-derived T_reg_ (nT_reg_) cells are generated in the thymus and are assumed to protect against the activity of autoreactive T cells in the periphery. These cells express the forkhead transcription factor FOXP3 and constitutively express CD25 on their surface, but they lack expression of Th1 or Th2 cytokines. Particularly interesting are those T_reg_ cells that are generated in the periphery and thus are potential targets for therapeutic interventions. These induced T_reg_ (iT_reg_) cells were reported to express FOXP3 [10]. The exact mechanisms of iT_reg_ generation are unclear, but T cell receptor (TCR) triggering has been shown to induce FOXP3 expression and suppressive cells in human [11,12], however the phenotype appears to be of transient nature [13,14]. TGF-β has been demonstrated to be important for the persistent induction of these cells in vitro and in vivo, since animals lacking the TGF-βRII on T cells have fewer peripherally iT_reg_ cells [15] and suffer from a T cell–dependent multiorgan inflammatory disease [16]. Although the effect of TGF-β on natural and inducible T_reg_ cell induction has been demonstrated repeatedly [15,17], its molecular mechanisms remain to be identified.

The current study provides evidence that GATA3 and FOXP3 play a competitive role in iT_reg_ cell commitment as T-bet and GATA3 for Th1 and Th2 differentiation, respectively. We show that GATA3 inhibits FOXP3 induction and that IL-4 limits FOXP3 expression in a GATA3-mediated way, both in vitro and in vivo. We also show that GATA3 directly binds to the FOXP3 promoter and thereby prevents the induction of this gene, demonstrating that Th2 differentiation overrules iT_reg_ induction.

## Results

### FOXP3 Induction in T Cell Subsets

**Figure 1:**
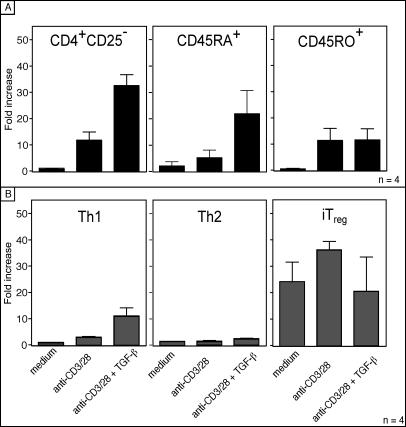
Th2 Cells Cannot Induce FOXP3 Expression (A) Human T cells were activated with plate-bound anti-CD3/CD28 with or without TGF-β as indicated on the *x*-axis of (B). Cells were harvested after 5 days and FOXP3 mRNA was quantified by real-time PCR. Bars show the mean ± SD of 4 independent experiments. (B) In vitro differentiated Th1, Th2, or iT_reg_ cells were activated with anti-CD3/CD28, TGF-β, or anti-IL-4 as indicated. The phenotype of these cells was confirmed by FACS and proliferation analysis (Figure S1). Bars show the mean ± SD of four independent experiments.

It is assumed that FOXP3 expression can be induced in nonregulatory T cells, which is an important step in iT_reg_ cell differentiation. However, it is not known if all CD4^+^ T cells have the same capacity to express FOXP3. To investigate whether FOXP3 can be expressed by any T cell subset or if expression is restricted to a distinct lineage, FOXP3 mRNA expression was analyzed in freshly isolated T cells such as CD25-depleted CD4^+^ cells, CD45RA^+^ naive or CD45RO^+^ memory T cells (Figure 1A), as well as T cells driven in vitro toward Th1, Th2, or iT_reg_ phenotypes (Figure 1B; phenotype on Figure S1). The CD4^+^CD25^–^, CD45RA^+^, CD45RO^+^, and CD4^+^CD45RO^+^CD25^–^ were able to significantly induce FOXP3 mRNA up to 30-fold upon TCR activation and addition of TGF-β. Th1 cells showed only a 10-fold increase. In contrast, Th2 cells stimulated under the same conditions did not increase FOXP3 expression. The in vitro generated iT_reg_ cells were unable to further up-regulate FOXP3, which was already at high levels under the resting conditions (Figure 1B, right panel).

**Figure 2:**
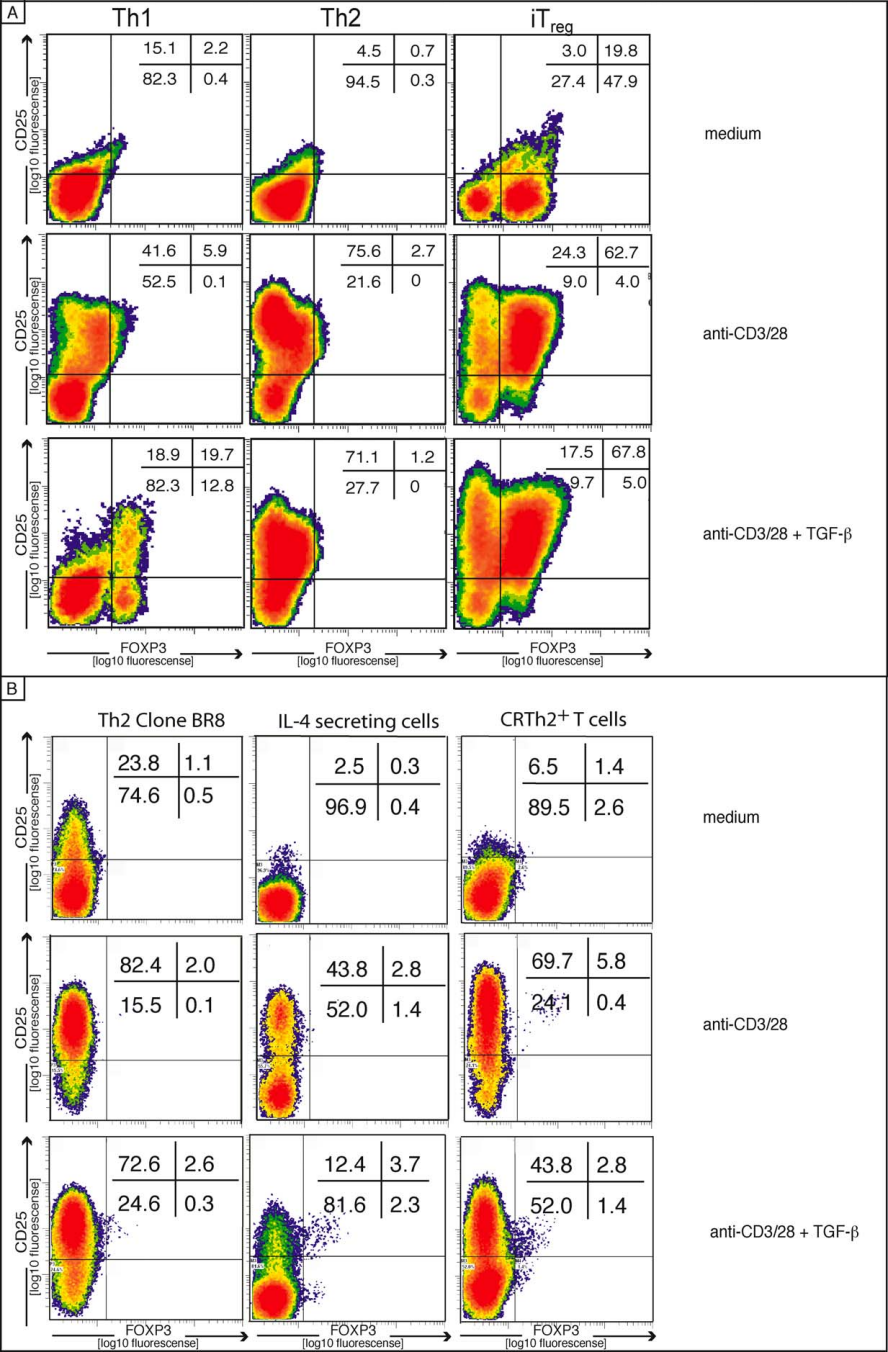
Th2 Cells Do Not Express FOXP3 (A) Intracellular FACS analysis of FOXP3 expression in Th1, Th2, or iT_reg_-differentiated cells (two rounds, phenotype see Figure S1), rested or activated, with or without TGF-β. FOXP3 expression was measured after 5 d in culture. The dot blots are representative of three independent experiments. (B) Shows the same experimental setup, but naturally occurring Th2 cells were analyzed. Data are representative of three independent experiments.

**Figure 3:**
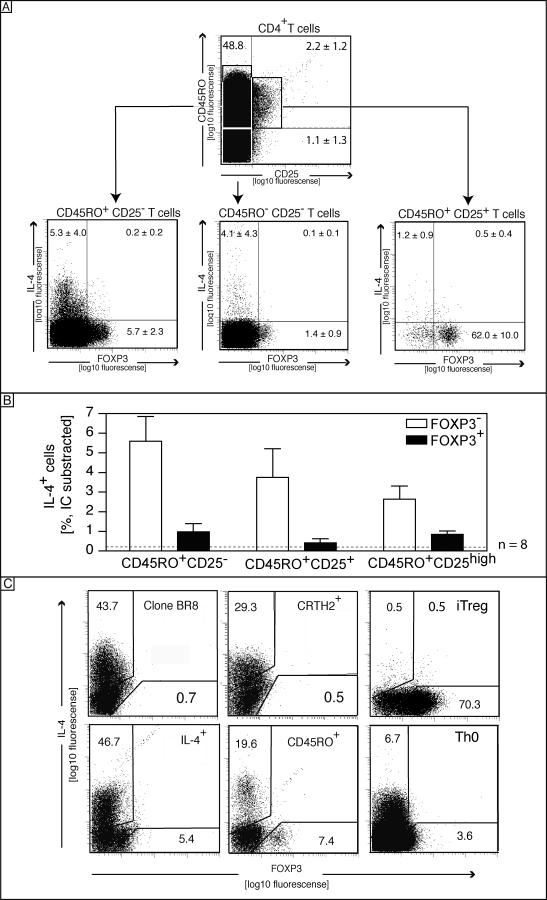
Th2- or IL-4–Producing Cells Lack FOXP3 (A) FACS analysis of intracellular FOXP3 and IL-4 expression following PMA/Ionomycin stimulation. CD4^+^ T cells were gated on the basis of CD45RO and CD25 surface expression (upper panel), and gated cells are shown below for the CD45RO^+^CD25^−^ (A, left panel), the CD45RO^+^CD25^+^ (right panel), and the CD45RO^−^CD25^−^ subsets (central panel). A statistical analysis of eight independent donors after subtraction of the isotype control are shown in (B). The dotted gray line indicates the IC background level. The error bars show the error of the mean. (C) Similarly, a Th2 clone (BR8), CRTH2^+^ Th2 cells, IL-4-secreting cells, and memory T cells (CD45RO) were stained for FOXP3 and IL-4. Data are representative of three independent experiments.

Th2 cells are known to produce IL-4 upon activation, which may interact with TGF-β signaling and thus prevent FOXP3 induction. However, the neutralization of IL-4 with a blocking IL-4 antibody did not rescue FOXP3 expression in the differentiated Th2 cells (unpublished data). These data demonstrated that Th2 cells have a limited capacity to express FOXP3 (Figure 1B). The inability of Th2 cells to express FOXP3 was also documented at the single-cell level, confirming that Th2 cells lack FOXP3 expression (Figure 2A). Only iT_reg_ cells expressed FOXP3 in resting conditions. Interestingly, we observed that resting iT_reg_ cells express FOXP3 but show low CD25 surface expression. Repeated exposure to TGF-β did not further increase the FOXP3 expression in the iT_reg_ lineage but transiently induced FOXP3 expression in Th1 cells. Naturally occurring Th2 cells such as CRTH2^+^ T cells, T cells isolated according to their IL-4 secretion, or an IL-4–producing T cell clone (BR8) were also lacking FOXP3 expression (Figure 2B). Furthermore TGF-β–mediated FOXP3 induction failed in these cells in contrast to the naive T cells (Figure 2B). Because IL-4 is the key Th2 cytokine, the expression of IL-4 and FOXP3 in freshly isolated CD4^+^ T cells was analyzed by fluorescence activated cell sorting (FACS). IL-4–expressing cells were most abundant among CD45RO^+^CD25^–^ cells, which did not co-express FOXP3 (Figure 3A, left panel). In contrast, CD45RO^+^CD25^+^ cells abundantly expressed FOXP3, while lacking IL-4 (Figure 3A, right panel). As shown in Figure 3B, the frequency of the IL-4^+^ cells was always below 1% in the FOXP3^+^ cells close to the background. The IL-4^+^ cells were confined to the FOXP3^–^ cells, as shown for the CD45RO^+^CD25^–^, CD45RO^+^CD25^+^, and CD45RO^+^CD25^+high^ cells (Figure 3B). In addition, neither the Th2 clone (BR8) nor CRTH2 cells significantly expressed FOXP3 (Figure 3C). Cells enriched for their IL-4 secretion using the magnetic cell isolation technology contained some FOXP3-expressing cells, but importantly, the expression did not overlap. Taken together, these data indicate that FOXP3 was not expressed by Th2 cells and was not inducible in those cells.

### FOXP3 and GATA3 Kinetics in Differentiating Cells

**Figure 4:**
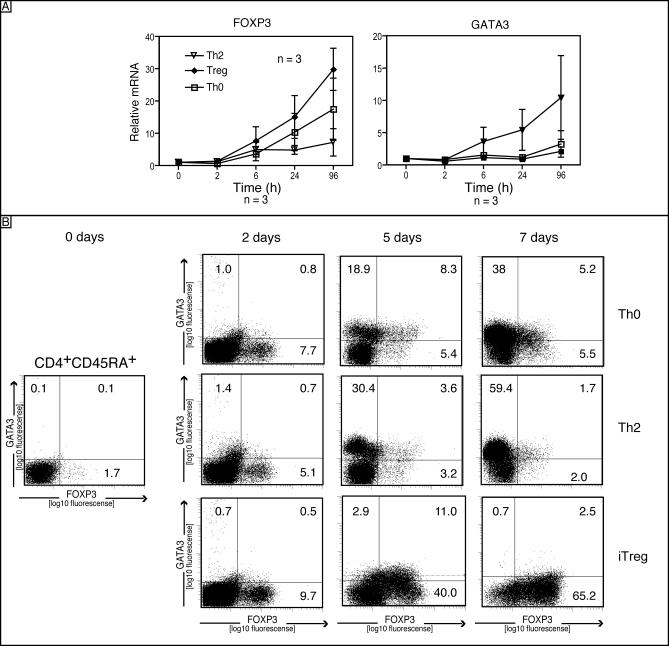
FOXP3 Induction During the Differentiation Process (A) Human CD4^+^CD45RA^+^ T cells were activated with plate-bound anti-CD3/CD28 in the presence of TGF-β (5 ng/ml) or IL-4 (25 ng/ml). The cells were harvested at different time points, and mRNA was quantified by real-time PCR for FOXP3 and GATA3 expression. Bars show the mean ± SD of three independent experiments*.* (B) Intracellular GATA3 and FOXP3 staining is shown after exposure of CD4^+^CD45RA^+^ T cells to differentiating conditions as in part A of the figure. Data are representative of three independent experiments.

The limited capacity of differentiated effector cells to induce FOXP3 expression suggests that iT_reg_ induction has to occur before effector T cell differentiation occurs. Therefore, we analyzed the expression of FOXP3 and GATA3 during the differentiation of naive CD4 T cells into Th0 (neutral, anti-IL-4, anti-IFN-γ, and anti IL-12), Th2, and iT_reg_ phenotypes. After initiation of the differentiation process, FOXP3 and GATA3 showed a similar expression kinetic within the first 3 d, which are considered to be critical in T cell commitment [18]. Under Th2 differentiation conditions, FOXP3 mRNA expression increased only marginally (Figure 4A, left panel). Thus, although GATA3 and FOXP3 showed similar kinetics, their expression polarizes at the end of the differentiation process when cells were cultured towards Th2 or iT_reg_ cells, respectively (Figure 4A and 4B). Interestingly, the Th0 cells were expressing more FOXP3 than the Th2 cells, but expressed low levels of GATA3; however, the protein expression slightly differed from mRNA expression, suggesting also posttranslational regulation and degradation as potential additional mechanisms in the differentiation process. The phenotype of iT_reg_ cells included an anergic phenotype upon anti-CD3 re-stimulation (Figure S1A), CD103, CTLA-4, GITR, and PD-1 surface expression (Figure S1B). On the single-cell level, it can be seen that cells progress through a transition phase, where GATA3 and FOXP3 expression coexist to some degree in the same cells, which is resolved in iT_reg_ cells after 7 d (Figure 4B). Taken together, these data demonstrated that Th2 cells have lost their capacity to express FOXP3 and showed that Th2 and iT_reg_ cells arise from two different differentiation pathways.

### IL-4 Inhibits TGF-β-Mediated iT_reg_ Commitment

**Figure 5:**
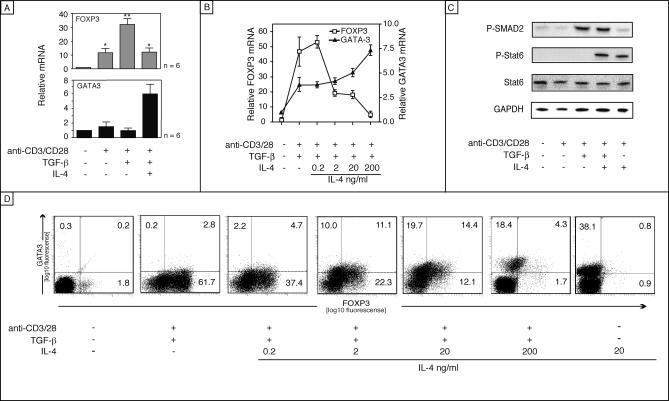
Effect of IL-4 on FOXP3 Induction (A) A statistical analysis was performed with six donors on day 5 (TGF-β (10 ng/ml) and with or without IL-4 (100 ng/ml)); Shown is the mean, and error bars indicated the SD of six donors. Statistical analysis was performed using the Dunnett test. Statistical significance is indicated by asterisks (*p ≤ 0.05, **p ≤ 0.01, Dunnett). (B) CD4^+^CD45RA^+^ cells were activated in the presence of a constant concentration of TGF-β (5 ng/ml) with an increasing concentration of IL-4, as indicated. Cells were harvested for mRNA quantification after 5 d. (C) CD4^+^CD45RA^+^ cells were stimulated in vitro with plate-bound anti-CD3/CD28, TGF-β (10ng/ml), and IL-4 (100 ng/ml) as indicated. After 1 h, cell lysates were prepared and analyzed by Western blot for phosphorylated SMAD2 and STAT6. Total STAT6 and GAPDH served as internal control. (D) Intracellular GATA3 and FOXP3 staining are shown after exposure of CD4^+^CD45RA^+^ T cells to IL-4 as described for panel B. Data are representative of three independent experiments.

IL-4 induces differentiation of naive T cells, upon antigen encounter, into the Th2 cell lineage. We therefore asked whether IL-4 is able to inhibit TGF-β induction of FOXP3 during the priming of naive T cells. Human CD4^+^CD45RA^+^ T cells were activated with plate-bound anti-CD3/CD28 in the presence of TGF-β and/or IL-4 and harvested after 5 d. IL-4 efficiently repressed the TGF-β–mediated induction of FOXP3 expression (Figure 5A) in a concentration-dependent manner (Figure 5B). Low levels of GATA3 were induced also in the absence of IL-4, as it was previously observed [3]. However, at low concentration, IL-4 was able to marginally induce FOXP3 expression. Of note, GATA3 was also induced in the presence of TGF-β at high IL-4 concentration (Figure 5A and 5B). The IL-4–mediated prevention of FOXP3 expression was not caused by interferences of the receptor signaling, because the phosphorylation of SMAD2 or STAT6 was not affected by the addition of IL-4 and/or TGF-β, which demonstrates that IL-4 as well as TGF-β signaling were functional under these conditions (Figure 5C). Increasing amounts of IL-4 increase intracellular GATA3, whereas FOXP3 decreased, which is consistent with the mRNA analysis (Figure 5D). Furthermore, injection of IL-4 into wild-type B6 mice decreased the inducible or natural T_reg_ number in vivo. A distinction of the T_reg_ subsets is not possible, because recently activated iT_reg_ cells also express surface CD25. We used complexes of recombinant mouse IL-4 (rmIL-4) plus anti-IL-4 monoclonal antibodies (mAbs), which have been shown to dramatically increase the potency of the cytokine in vivo [19]. In these mice, the percentage of CD4^+^CD25^+^ and FOXP3^+^ T cells dramatically decreased when the antibody-cytokine immune complexes were injected (Figure S2). Upon administration of rmIL-4 plus anti-IL-4 mAb complexes, the total number of CD4^+^CD25^+^ T cell, as well as the Foxp3^+^ T cells diminished by half (Figure S2G and S2H), confirming that the lower percentage was not due to an increase in the CD4^+^CD25^–^ cells, but a real decrease of CD4^+^CD25^+^ T cells. In conclusion, IL-4 negatively regulates the natural or inducible T_reg_ cell turnover not only in vitro but also in vivo. To study the effects of IL-4 on already-existing human natural or inducible T_reg_ cells, we exposed sorted CD25^+^ T cells (nT_reg_ cells) to IL-4 and analyzed FOXP3 expression and suppressive capacity. In already-existing T_reg_ cells, IL-4 failed to inhibit FOXP3 expression (Figure S3A), and the suppressive capacity was not altered (Figure S3C). Similarly pre-existing iT_reg_ cells did not decrease FOXP3 expression upon IL-4 exposure (Figure S3B).

### The Role of TGF-β in T Cell Differentiation

**Figure 6:**
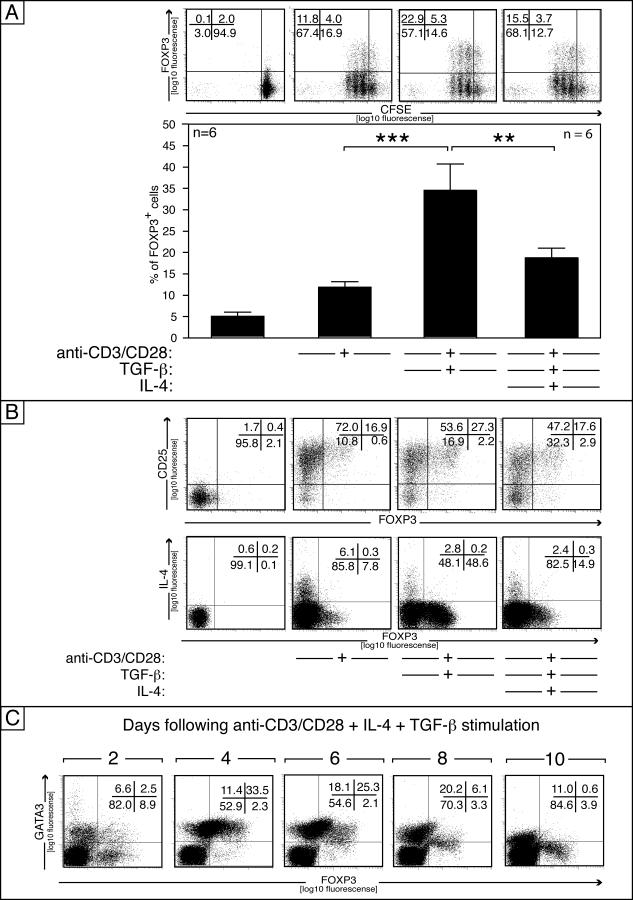
IL-4 Inhibits TGF-β–Mediated iT_reg_ Commitment CFSE-labeled CD4^+^CD45RA^+^ cells were activated with plate-bound anti-CD3/CD28, TGF-β, and IL-4, as indicated. After 5 d, cells were analyzed by flow cytometry (A) and results of six independent experiments are shown in the bar graph below (B). Statistical significance (one-way Anova, Newman-Keuls) is indicated by asterisks (**p ≤ 0.01, ***p ≤ 0.001). (C) Kinetic analysis of intracellular GATA3 and FOXP3 staining is shown in panel C following exposure of CD4^+^CD45RA^+^ T cells to anti-CD3/28, IL-4 and TGF-β. Data are representative of three independent experiments.

Although TGF-β–reduced CD25, IL-4 expression, and CD25 expression (Figure 6B), IL-4 significantly inhibited TGF-β–mediated induction of FOXP3 in naive T cells driven toward FOXP3^+^ T cells, as shown by FACS analysis (Figure 6A). It is known that IL-4 is a potent growth factor and may therefore favor the proliferation of FOXP3^–^ cells and thus decrease the relative percentage of FOXP3^+^ cells. However, analysis of cell division kinetics by CFSE-labeling demonstrated that IL-4 did not differentially promote cell growth of FOXP3^+^ over that of FOXP3^–^. In fact both populations showed similarly enhanced proliferation (Figure 6A). Furthermore the TGF-β–mediated induction of FOXP3 expression was not caused by overgrowth of a CD25^–^FOXP3^+^ minority, since the number of FOXP3^+^ cells was low/absent in the purified CD4^+^CD45RA^+^ T cells (between 0% and 1%), and the FOXP3^+^ cells were not confined to the highly divided cells. CD25 was down-regulated in TGF-β–treated cells compare to activated T cells, which was even more pronounced in cells cultured with TGF-β and IL-4.

The addition of IL-4 to iT_reg_-driving conditions decreased the number of FOXP3^+^ cells (Figure 6B). In line with the previous findings, the IL-4–producing cells and the FOXP3 expressing cells are nonoverlapping populations. Since FOXP3 is known to act as a repressor of cytokine expression [20], we therefore analyzed GATA3 and FOXP3 expression. The expression kinetic of naive T cells exposed to IL-4 and TGF-β demonstrated that GATA3 and FOXP3 are initially found in separate populations (day 2), but transiently co-express both factors (days 4–8), before establishing separate populations at the end of the differentiation process (day 10; Figure 6C), suggesting that GATA3 inhibits the development of iT_reg_ cells by repressing FOXP3.

These results showed that IL-4 acts in vitro as an inhibitor of FOXP3 expression, without interfering with TGF-β signaling, probably acting at the level of transcription factors, and possibly by a GATA3-dependent mechanism.

### GATA3 Is a Negative Regulator of FOXP3 Expression

**Figure 7:**
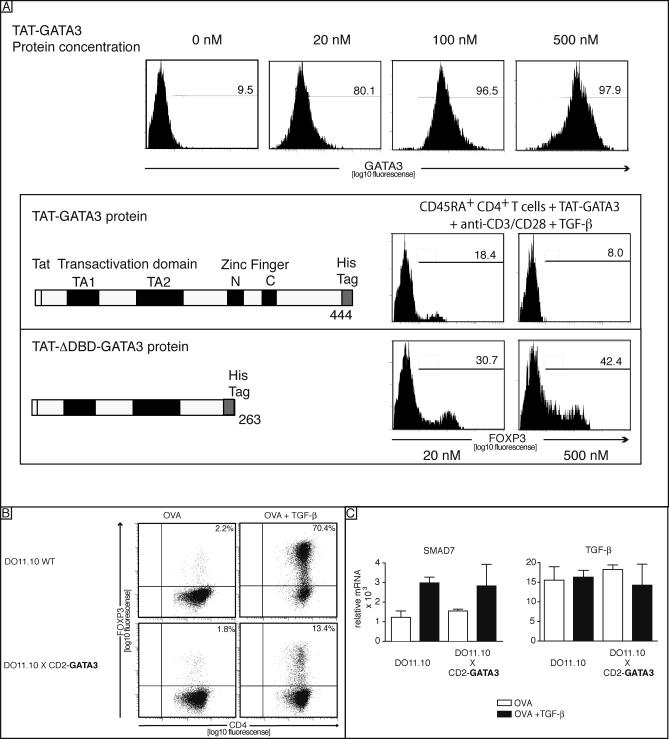
GATA3 Acts as a Negative Regulator of FOXP3 Expression (A) Human naive CD4^+^CDRA^+^ T cells were transduced with 0, 20, 100, and 500 nM of TAT-GATA3 protein, and intracellular presence of GATA3 was analyzed using FACS following anti-CD3/CD28 activation of the cells. GFP-positive cells were gated and analyzed for intracellular FOXP3 expression following a 2-d incubation period (lower panel). Data are representative of four independent experiments. (B) CD4^+^CD25^−^ T cells were isolated from D011.10 and D011.10xCD2-GATA3 mice and treated with OVA and TGF-β for 96 h. Surface CD4 and intracellular FOXP3 were measured by FACS. These data are representative of three independent experiments. (C) The cells treated as in (B) were harvested and mRNA was quantified by real-time PCR for SMAD7 and TGF-β expression. Bars show the mean ± SD of three independent experiments.

FOXP3 expression decreased once GATA3 expression is high; therefore, we hypothesized a potential role for GATA3 in repressing FOXP3. Besides GATA3′s well-known positive effect on gene regulation, GATA3′s repressive capabilities were previously shown to restrict Th1 commitment by inhibiting STAT4 expression [2,21], and therefore GATA3 prevents differentiation into Th1 cells. To investigate whether GATA3 can directly inhibit FOXP3 induction, we transduced GATA3 or a truncated GATA3 lacking the DNA-binding domain in human primary CD4^+^CD45RA^+^ T cells using a TAT-fused, recombinantly expressed GATA3. After transduction, the cells were activated with soluble anti-CD3/CD28 in the presence or absence of TGF-β. TAT-GATA3 was successfully transduced in a homogeneous and dose-dependent manner into human CD4^+^ T cells (Figure 7A, upper panel). TAT-GATA3 reduced FOXP3 expression in a dose-dependent manner, whereas a DNA-binding domain truncated version (TAT-ΔDBD-GATA3) did not affect FOXP3 expression as compared with expression in untransduced cells (Figure 7A). In addition, we analyzed the inhibitory effect of GATA3 on FOXP3 in transgenic DO11.10 mice, constitutively overexpressing GATA3 under the control of the CD2 locus control region (DO11.10xCD2-GATA3). The thymic selection into the CD4 lineage is largely intact in DO11.10xCD2-GATA3 (RW Hendriks, unpublished data). These mice develop lymphomas at an older age, but signs of autoimmune disease were not described [22]. To investigate the effect of GATA3 on iT_reg_, CD4^+^CD62L^+^CD25^–^ cells were isolated, activated with OVA in the presence or absence of TGF-β, and Foxp3 expression was analyzed after 4 d. The naive CD4^+^CD25^–^ cells were Foxp3^–^ (unpublished data). As described for the human cells, TGF-β dramatically up-regulated Foxp3 in the DO11.10 littermate control mice. In contrast, cells from the CD2-GATA3xDO11.10 mice showed dramatically reduced Foxp3 expression when activated with TGF-β and OVA (Figure 7B). All mice produced similar amounts of TGF-β; in addition, Smad7 was equally expressed [23] in T cells of both mice strains (Figure 7C), indicating intact TGF-β signaling.

Taken together, these results demonstrated a repressive role of IL-4-induced GATA3 transcription factor in the generation of iT_reg_ cells.

### GATA3 Represses the FOXP3 Promoter

**Figure 8:**
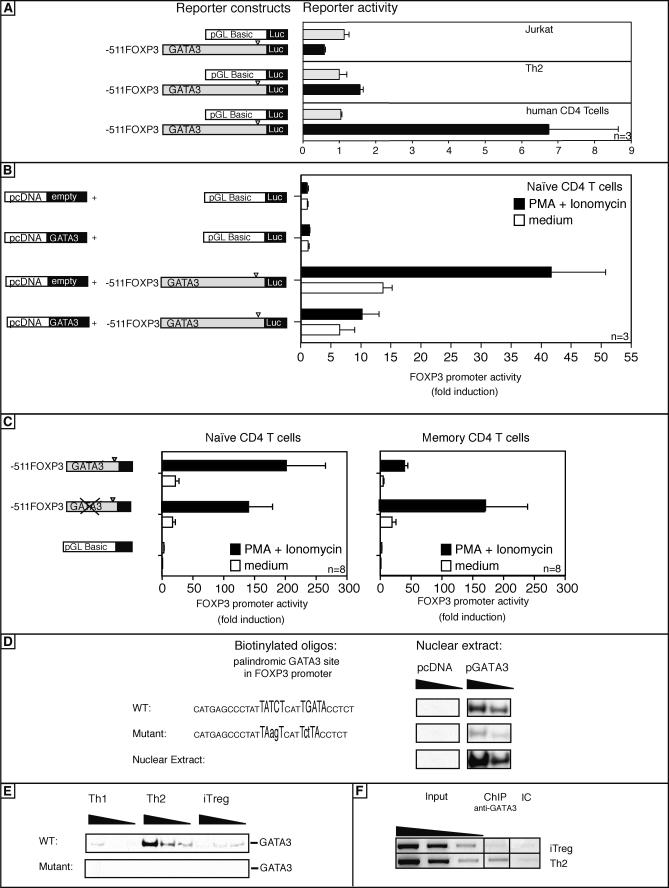
GATA3 Represses the Human FOXP3 Promoter (A) Jurkat, Th2 cells, and human primary CD4 cells were transfected with an empty vector (pGL3 basic) or a vector containing the putative FOXP3 promoter region fused to the luciferase reporter gene. Bars show the mean ± SD of arbitrary light units normalized for renilla luciferase of four independent experiments; samples were measured in triplicates. (B) Naive CD4 T cells were transfected with the FOXP3 promoter reporter construct together with a GATA3 expression vector or an empty vector. Bars show the mean ± SD of three independent experiments. (C) Naive (left panel) or memory (right panel) CD4 T cells were transfected with wild-type or a GATA3 mutated 511-FOXP3 promoter reporter construct and activated with PMA and ionomycin. Bars show the mean ± SD of arbitrary light units normalized for renilla luciferase of eight independent experiments; samples were measured in triplicates. (D) Nuclear extracts were prepared from HEK cells transfected with GATA3 or an empty vector, (E) Th1, Th2, or iT_reg_ cells and binding factors precipitated using biotinylated oligonucleotides. The oligonucleotides–transcription factor complexes were separated on a SDS-PAGE gel. The amounts of GATA3 protein in the precipitates were assessed by immunoblotting with anti-GATA3 mAb. Total nuclear extracts were also run as controls. Data are representative of three different experiments. (F) iT_reg_ or Th2 cells were analyzed by ChIP for GATA3 binding to the FOXP3 promoter. The “input” represents PCR amplification of the total sample, which was not subjected to any precipitation. Results are representative of three independent experiments.

To investigate the molecular mechanism of GATA3-mediated repression of human FOXP3, the human FOXP3 promoter was studied and a palindromic binding site for GATA3 was discovered. The GATA-binding site is located 303 bp upstream from the transcription start site (TSS) [24]. This site is highly conserved between humans, mice, and rats (Figure S4) and may therefore play an important role in FOXP3 regulation. The functional relevance of this site was studied using a FOXP3-promoter construct [24]. We transfected human primary CD4^+^ T cells, in vitro differentiated Th2 cells, and Jurkat cells (Jurkat cells are known to constitutively express GATA3 [25,26]), and we measured FOXP3 promoter activity. The promoter was not active in the GATA3-expressing cell line Jurkat or in the in vitro-differentiated Th2 cells, whereas the construct was active in the CD4 cells, which express a lower amount of GATA3 (Figure 8A). Overexpression of GATA3 in naive T cells diminished luciferase activity of the FOXP3 promoter compared with the control vector (Figure 8B). To further address the function of the GATA3 site, we inserted a site-specific mutation deleting the GATA3-binding site. This mutation increased luciferase activity by 3-fold in memory CD4^+^CD45RO^+^ T cells, whereas no difference was observed in naive (GATA3^–^) CD4^+^CD45RA^+^ T cells, revealing a repressor activity of GATA3 on the FOXP3 promoter (Figure 8C). Furthermore GATA3 binds directly to the FOXP3 promoter as investigated by pull-down assay. HEK cells were transiently transfected with GATA3 or a control vector, and increasing amounts of lysates were incubated with oligonucleotides containing the GATA3 site of the FOXP3 promoter or a control oligonucleotide with a mutated GATA3-binding site. After the pull-down, GATA3 binding was detected by Western blot. Similarly, GATA3-expressing Th2 cells and iT_reg_ cells were subjected to this approach. Only HEK cells overexpressing GATA3 and Th2 cells showed GATA3-binding activity (Figure 8D and 8E). These experiments demonstrated that GATA3 binds the palindromic FOXP3 promoter. To gain insights into the in vivo situation, we performed a chromatin immunoprecipitation (ChIP) using an anti-GATA3 antibody and showed that GATA3 binds to the FOXP3 promoter region in Th2 cells, but not in iT_reg_ cells (Figure 7F). Taken together these data demonstrate that the GATA3-binding to the FOXP3 promoter is repressing FOXP3 expression.

## Discussion

The current study reveals that FOXP3 induction, an important step in iT_reg_ commitment, is inhibited by GATA3, which is the key regulator for polarization toward Th2 cells. After differentiation, the effector Th2 cells become refractory to conversion into a FOXP3^+^ phenotype.

In accordance with other studies, we found that CD4^+^CD25^–^ cells were able to up-regulate FOXP3 [12,27]. Already-committed cells such as memory T cells and Th1 cells showed only moderate and transient FOXP3 induction, which is not sufficient to change the phenotype toward a regulatory T cell profile. In contrast, naive T cells could efficiently up-regulate FOXP3 when treated with TGF-β to induce iT_reg_ cells [10,28–34], suggesting that FOXP3 plays an important role in the early differentiation process and may act in a way similar to that known for the Th1/Th2 decision factors T-bet and GATA3. This commitment is characterized by competitive expression of these factors [35,36], which we also observed in differentiating FOXP3^+^ iT_reg_ cells including a phase of co-expression, which turns into nonoverlapping expression upon completed differentiation. In this competitive process TGF-β appeared to be mandatory for the induction of FOXP3, possibly by keeping the expression of GATA3 and T-bet low [37,38]. In contrast, differentiating naive T cells in the absence of polarization factors (Th0) such as IL-4, IL-12, or TGF-β showed only a transient FOXP3 expression and failed to generate a population of FOXP3-expressing cells, but GATA3 and T-bet were up-regulated (unpublished data). Interestingly, as we and others previously described, FOXP3-promoting factors, such as dexamethasone [39], CTLA-4 [40], and estrogens [41], are also known as inhibitors of GATA3 expression [42–45]. Therefore GATA3 not only induces differentiation into Th2 cells but also inhibits FOXP3 expression and commitment into iT_reg_ cells.

The Th2 cytokine IL-4 but not IL-13 (unpublished data) was able to inhibit TGF-β–mediated FOXP3 induction and therefore prevented conversion into the regulatory phenotype. To prove the inhibitory effect of IL-4 on inducible or natural T_reg_ commitment in vivo, we treated mice with IL-4 and anti-IL-4. This has been shown to increase the effect of the cytokine in vivo [19]. Only the IL-4/IL-4 mAb complex resulted in a decrease of the amount of natural or inducible T_reg_ (CD25^+^ and Foxp3^+^) cells 7 d after treatment. Our results suggest that IL-4 is only interfering with the differentiation of naive T cells into iT_reg_ cells. But since a distinction of nT_reg_ and iT_reg_ cells is currently not possible, because iT_reg_ cells also transiently express CD25 after activation, we cannot exclude that IL-4 may also inhibit Foxp3 expression in nT_reg_ cells in vivo or that additional effects may contribute to the observed drop in Foxp3 expression. IL-4 has already been shown to negatively regulate the development of naive T cells into Th1 or the IL-17–producing T cells (Th17) [46,47]. Similar effects have been recently described for IL-6, which, combined with TGF-β, inhibits the generation of iT_reg_ cells and induces differentiation into the Th17 cells by an unknown mechanism [48,49]. Thus the polarization into iT_reg_ cells is negatively regulated by the effector cytokines IL-4 and IL-6.

IL-4 has been previously shown to induce the generation of FOXP3^+^ T_reg_ cells out of CD4^+^CD25^–^ [50]. In those experiments, the concentrations of IL-4 used were low, and as we also observed, IL-4 at low concentration slightly enhanced FOXP3 expression. Importantly, these concentrations were not sufficient to induce GATA3 expression. IL-4 may favor proliferation of nT_reg_ cells [47] or directly regulate FOXP3 expression in a STAT-dependent fashion [51].

Since IL-13 does not effectively reduce FOXP3 and fails to induce GATA3, we hypothesized that the IL-4–dependent inhibition of FOXP3 could be mediated by GATA3. In fact, GATA3-inducing IL-4 concentrations repressed TGF-β–mediated FOXP3 expression, whereas IL-4 as well as TGF-β signaling were intact. This result suggested a competitive mechanism between GATA3 and FOXP3 transcription factors in determining lineage commitment during the early phase of differentiation. Accordingly, we investigated naturally high GATA3-expressing cells and confirmed the absence of FOXP3. Protein transduction of GATA3 into naive T cells inhibited FOXP3 induction in human, differentiating, naive T cells. This inhibitory effect of GATA3 was further confirmed in BALB/c transgenic mice, expressing GATA3 in T cells (DO11.10:CD2-GATA3 transgenic mice). In line with the transient overexpression of GATA3 in human T cells, cells of these mice failed to induce FOXP3 expression upon exposure with antigen in the presence of TGF-β. Strikingly, the DO11.10:CD2-GATA3 mice do have peripheral FOXP3^+^ cells, which however displayed a 10%–25% lower frequency compared to wild-type DO11.10 mice. Thus GATA3 restrains the development of certain T_reg_ subsets, presumably the inducible, peripheral population and not those of thymic origin. Thymic T cells undergo a different maturation process, which may explain the insensitivity of nT_reg_ to GATA3 overexpression [52]. In contrast to the Th2-differentiating and iT_reg_-inhibiting function of GATA3 in peripheral T cells, GATA3 acts in the thymus together with other transcription factors such as the *Repressor of GATA3* (ROG) in the differentiation process toward CD8 cells [53,54] or participates in complex transcriptional feedback network to regulate sympathoadrenal differentiation [55]. Therefore, the role of GATA3 appears to be tissue specific and cannot be generalized.

Our study demonstrates that GATA3 repressed FOXP3 expression directly by binding to the FOXP3 promoter region. A palindromic GATA-site is located 303 bp upstream of the TSS in a highly conserved region, which we have previously identified as the FOXP3 promoter [24]. Site-specific mutation of this site increased the activity of the promoter constructs, thus revealing the repressive nature of this GATA element in memory T cells, which naturally express GATA3, whereas no difference was seen in naive T cells, which do not express GATA3. This palindromic GATA element is bound by GATA3 protein as proven with pull-down experiments. Furthermore, it is shown by ChIP that GATA3 binds this element also in intact cells. It is known that GATA3 can induce transcription by chromatin remodeling [56], by directly transactivating promoters [36], or, as shown in the current study, acts as a repressor of gene expression [21,57–59]. Therefore keeping GATA3 expression low might be required to induce efficient FOXP3^+^ iT_reg_ cell generation.

The molecular interactions enabling GATA3 to inhibit FOXP3 are not identified yet, but the GATA-binding site is located adjacent to positive, inducing sites, composed of AP-1-NFATc2 sites [24], and GATA3 may compete with the binding of AP-1/NFAT to the promoter (unpublished observations).

In summary, we demonstrated that FOXP3 is negatively regulated by cytokines such as IL-4. GATA3 acts as an inhibitor of FOXP3 expression in early T cell differentiation, as well as in differentiated Th2 cells by directly binding and repressing the FOXP3 promoter. We therefore describe a new mechanism of how Il-4 avoids tolerance induction by repressing FOXP3 expression. These findings will give new perspectives toward understanding molecular mechanisms of iT_reg_ induction and thus pathways of peripheral tolerance induction, particularly in allergy and asthma.

## Materials and Methods

### Mice.

Normal B6 mice were purchased from the Jackson Laboratories (Bar Harbor, Maine). Transgenic DO11.10 mice, expressing a T cell receptor for OVA_323–339_ peptide in the context of H-2^d^, were backcrossed with mice expressing GATA-3, driven by the human CD2 locus control region (CD2-GATA3) [22], resulting in DO11.10xCD2-GATA3 mice. Mice used for experiments were backcrossed on a BALB/c background for a minimum of eight generations and used at an age of 8–12 wk. Mice were housed under specific pathogen-free conditions and all animal studies were performed according to institutional and state guidelines.

### Isolation of CD4^+^ T cells.

CD4^+^ T cells were isolated from blood of healthy human volunteers using the anti-CD4 magnetic beads (Dynal, Hamburg, Germany) as previously described [60]. The purity of CD4^+^ T cells was initially tested by FACS and was ≥ 95%. Monoclonality of T cell clones was confirmed by TCR-chain mapping and was identified to be Vbeta8 positive. The clones were characterized by high IL-4 secretion.

### Quantitative real-time PCR.

The PCR primers and probes were designed based on the sequences reported in GenBank with the Primer Express software version 1.2 (Applied Biosystems) as follows: FOXP3 forward primer 5′-GAA ACA GCA CAT TCC CAG AGT TC-3′; FOXP3 reverse primer 5′-ATG GCC CAG CGG ATG AG-3′; EF-1α forward primer and reverse primer as described [61]; GATA3 forward primer 5′-GCG GGC TCT ATC ACA AAA TGA-3′ and rwd 5′-GCT CTC CTG GCT GCA GAC AGC-3′. The prepared cDNAs were amplified using SYBR-PCR mastermix (Biorad) according to the recommendations of the manufacturer in an ABI PRISM 7000 Sequence Detection System (Applied Biosystems).

Quantitative PCR of murine samples was performed with Brilliant SYBR Green QPCR master mix (Stratagene) and the following primers: Ubiquitin C, 5′- AGG TCA AAC AGG AAG ACA GAC GTA-3′ and 5′-TCACACCCAAGAACAAGCACA-3′; Smad-7, 5′-GAA ACC GGG GGA ACG AAT TAT-3′ and 5′-CGC GAG TCT TCT CCT CCC A-3′; TGF-ß_1_, 5′-TGA CGT CAC TGG AGT TGT ACG G-3′ and 5′-GGT TCA TGT CAT GGA TGG TGC-3′. Primer pairs were evaluated for integrity by analysis of the amplification plot, dissociation curves, and efficiency of PCR amplification. PCR conditions were 10 min at 95 °C, followed by 40 cycles of 15 s at 95 °C and 60 °C for 1 min using an 7300 real-time PCR system (Applied Biosystems). PCR amplification of the housekeeping gene encoding ubiquitin C was performed during each run for each sample to allow normalization between samples. Relative quantification and calculation of the range of confidence was performed using the comparative ΔΔCT method.

### Inducible murine T_reg_ culture.

Naive CD4^+^ T cells (CD4^+^, CD62L^+^, and CD25^–^) were isolated from pooled lymph nodes and spleens by FACS (FACS Aria, BD Biosciences). 5 × 10^5^ T cells were co-cultured with 2.5 × 10^4^ bone marrow–derived dendritic cells [62] and 0.01 μg/ml OVA_323–339_ peptide (Ansynth) in the presence or absence of 20-ng/μl rhTGF-ß_1_ (Peprotech) in 48-well plates. After 4 d, cells were harvested and analyzed for intracellular FOXP3 expression by FACS or gene expression by quantitative RT-PCR.

### In vitro T cell differentiation.

CD4^+^ CD45RA^+^ magnetically-sorted (CD45RO depletion, MACS, according to the protocol of the manufacturer) cells were stimulated with immobilized plate-bound anti-CD3 (1 μg/ml, Okt3, IgG1) and anti-CD28 (2 μg/ml) in Th1 conditions: 25 ng/ml IL-12, 5 μg/ml anti-IL-4 (R&D systems); in Th2 conditions: 25 ng/ml IL-4, 5 μg/ml anti-IFN-γ, 5 μg/ml anti-IL-12 (R&D systems); or in T_reg_ conditions: 10 ng/ml TGF-β, 5 μg/ml anti-IFN-γ, 5 μg/ml anti-IL-12, 5 μg/ml anti-IL-4. Proliferating cells were expanded in medium containing IL-2 (30 ng/ml).

### Cloning of the FOXP3 promoter and construction of mutant constructs.

The FOXP3 promoter was cloned into the pGL3 basic vector (Promega Biotech) to generate the pGL3 FOXP3 −511/+176 [24]. Site-directed mutagenesis in the FOXP3 promoter region were introduced using the QuickChange kit (Stratagene), according to the manufacturer's instructions. The following primer and its complementary strand were used: GTT TCT CAT GAG CCC TAT TAA GTC ATT CTT ACC TCT CAC CTC TGT GGT GA.

### Transfections and reporter gene assays.

T cells were rested in serum-free AIM-V medium (Life Technologies) overnight. 3.5 μg of the FOXP3 promoter luciferase reporter vector and 0.5 μg phRL-TK were added to 3 × 10^6^ CD4^+^ T cells resuspended in 100 μL of Nucleofector solution (Amaxa Biosystems) and electroporated using the U-15 program of the Nucleofector. After a 24-h culture in serum-free conditions and stimuli as indicated in the figures, luciferase activity was measured by the dual luciferase assay system (Promega Biotech) according to the manufacturer's instructions. Data were normalized by the activity of renilla luciferase.

### RNA isolation and cDNA synthesis.

RNA was isolated using the RNeasy Mini Kit (Qiagen) according to the manufacturer's protocol. Reverse transcription of human samples was performed with TaqMan reverse-transcription reagents (Applied Biosystems) with random hexamers according to the manufacturer's protocol.

### Recombinant TAT proteins.

The cDNAs encoding GATA3 protein or the truncated GATA3 (lacking the two zinc fingers) were cloned in frame into an expression vector along with the TAT sequence as previously described [63]. Proteins were expressed in BL21 Star (DE3)pLysS (Invitrogen) and lysates were purified by Ni^2+^-chelate column chromatography. Both TAT-linked proteins were more than 95% pure, based on Coomassie blue staining of sodium disulfate acrylamide gels.

### TAT-GATA3 transduction.

CD4^+^CD45RA^+^ cells were cultured in AIMV medium and transduced with 20 nM, 10 nM, or 500 nM of full-length or truncated GATA3 over the course of 4 h. After 4 h, the cells were washed and activated with soluble anti-CD3 and anti-CD28 and TGF-β (10 ng/ml). Each day, the TAT proteins were freshly added to the medium. FOXP3 expression was measured after 5 d by intracellular staining.

### Intracellular cytokine staining.

T cells were stimulated with 2 × 10^−7^ M PMA and 1 μg/ml of ionomycin (Sigma Chemicals) for 4 h. The following mAb was used: anti-IL-4-PE (8D4–8, BD). Matched isotype controls were used at the same protein concentration as the respective antibodies. Four-color FACS was performed using an EPICS XL-MCL (Beckman Coulter) using the software Expo32 version for data acquisition and evaluation.

### Flow cytometry.

For analysis of FOXP3 expression at the single-cell level, cells were first stained with the monoclonal antibody CD25 (Beckman Coulter), and after fixation and permeabilization, cells were incubated with PE-conjugated monoclonal antibody PCH101 (anti-human FOXP3; eBioscience) based on the manufacturer's recommendations and subjected to FACS (EPICS XL-MCL). For cell surface marker staining, cells were incubated for 20 min at 4°C in staining buffer with the following antibodies: anti–CD152-PE (CTLA-4; BD), anti–PD-1 (eBiosciences), anti-GITR (R & D Systems), anti-CD69 (Beckman Coulter), anti-CD103 (DakoCytomation), anti-CD62L (Beckman Coulter), or anti-HLA-DR (Beckman Coulter). The controls were FITC, PE, or ECD-conjugated mouse IgG1 or rat IgG2a. For staining of mouse cells, the following mAbs from BD Biosciences were used following standard techniques as described above: anti-CD3, anti-CD4, and anti-CD25. Anti-FcγRII/III antibody (2.4G2, ATCC) was included in all stainings to reduce nonspecific antibody binding. To isolate naive murine CD4 T cells from murine DO11.10 or DO11.10xCD2-GATA3 T cells, cells were stained with anti-CD25-FITC, anti-CD62L-PE, and anti-CD4-APC prior to sorting. Dead cells were excluded with 4′',6-Diamidino-2-phenylindole (DAPI). To analyze murine Foxp3 expression in inducible T_reg_ cultures, cells were stained intracellularly with anti-Foxp3-PE according to manufacturer's instruction, in conjunction with anti-CD4-APC and LIVE/DEAD fixable dead cell stain kit (Invitrogen) to discriminate live cells. All monoclonal antibodies for murine cell stainings were purchased from eBioscience or BD Biosciences.

### Western blotting.

For FOXP3 analysis on the protein level, 1 × 10^6^ CD4^+^CD25^–^ cells were lysed and loaded next to a protein-mass ladder (Magicmark, Invitrogen) on a NuPAGE 4–12% bis-tris gel (Invitrogen). The proteins were electroblotted onto a PVDF membrane (Amersham Life Science). Unspecific binding was blocked with BSA, and the membranes were subsequently incubated with an 1:200 dilution of goat anti-FOXP3 in blocking buffer (Abcam) overnight at 4 °C. The blots were developed using an anti-goat HRP-labeled mAb (Amersham Biosciences) and visualized with a LAS 1000 camera (Fuji). Membranes were incubated in stripping buffer and re-blocked for 1 h. The membranes were re-probed using anti-GATA3 (HG3–31; Santa Cruz Biotechnology), anti-T-bet (4B10, Santa Cruz Biotechnology), anti-GAPDH (6C5, Ambion), anti-phospho-SMAD2 (138D4), anti-phospho-STAT6 (5A4), and anti-STAT6 (Cell Signaling Technology),

### Administration of cytokines and antibodies in vivo.

Age- and gender-matched normal B6 mice received every other day intraperitoneal (ip) injections of PBS, 1.5 μg rmIL-4, 50 μg anti-IL-4 mAb (11B11 or MAB404), or a mixture of 1.5 μg rmIL-4 plus 50 μg anti-IL-4 mAb (11B11 or MAB404) for 7 d. Thereafter, spleen and lymph node cells were analyzed by flow cytometry for CD3, CD4, and CD25 expression. The anti-mouse IL-4 mAb MAB404 was obtained from R&D Systems, the second anti-mouse IL-4 mAb 11B11 was purchased from eBioscience.

### ChIP.

ChIP analysis was performed according to the manufacturer's protocol (Upstate Biotechnology) with the following modifications. iT_reg_ and Th2 cells were fixed with 1% formaldehyde for 10 min at room temperature. The chromatin was sheared to 200-1000 bp of length by sonication with five pulses of 10 s at 30% power (Bandelin). The chromatin was pre-cleared for 2 h with normal mouse IgG beads and then incubated with anti-GATA3-agarose beads (HG3–31; Santa Cruz Biotechnology) for 2 h. Washing and elution buffers were used according to the protocol of Upstate Biotechnology. Crosslinks were reversed by incubation at 65 °C for 4 h in the presence of 0.2 M NaCl, and the DNA was purified by phenol/chloroform extraction. The amount of DNA was determined by conventional PCR. The PCR addressed for the FOXP3 promoter region −246 to −511 and was performed using the following primers: 5′-gtgccctttacgagt catctg-3′ and 5′-gtgccctttacgagtcatctg-3′. The PCR products were visualized using an ethidium bromide gel.

### Pull-down assay.

CD4^+^ T cells were stimulated with PMA and ionomycin for 2 h at 37°C. The cells were pelleted, resuspended in buffer C (20 mM HEPES [pH 7.9], 420 mM NaCl, 1.5 mM MgCl_2_, 0.2 mM EDTA, 1 mM DTT, protease inhibitors [Sigma]. and 0.1% NP-40) and lysed on ice for 15 min. Insoluble material was removed by centrifugation. The supernatant was diluted 1:3 with buffer D (as buffer C, but without NaCl). The lysates were incubated with 10 μg of poly(dI-dC) (Sigma) and 70 μl of streptavidin-agarose (Amersham Biosciences) carrying biotinylated oligonucleotides, for 3 h at 4 °C. The beads were washed twice with buffer C:D (1:3) and resuspended in DTT-containing loading buffer (NuPAGE; Invitrogen), heated to 70 °C for 10 min, and the eluants on a NuPAGE 4–12% bis-tris gel (Invitrogen). The proteins were electroblotted onto a PVDF membrane (Amersham Biosciences) and detected using an anti-GATA3 mAb (Santa Cruz Biotechnology). Accumulated signals were analyzed using AIDA software (Raytest).

## Supporting Information

Figure S1 Phenotype of In Vitro Differentiated T Cells After two round of differentiation cultures, T cells were stimulated by plate-immobilized anti-CD3/CD28 and ^3^H-thymidine incorporation as measurement of proliferation was analyzed after 3 d of culture (A). In parallel, T cells were analyzed for T_reg_ relevant surface receptor expression as indicated on the x-axis (B).
(1.0 MB AI).

Figure S2 In Vivo Treatment of Mice with IL-4 Antibody–Cytokine Complexes B6 mice were given every other day ip injections of phosphate-buffered saline (PBS), recombinant mouse IL-4 (rmIL-4), anti-IL-4 mAb (anti-IL-4 mAb, 11B11, or MAB404), or a mixture of rmIL-4 plus anti-IL-4 mAbs (11B11 or MAB404). Mice were analyzed on day 7 by flow cytometry for CD3, CD4, and CD25 expression. Shown is CD25 versus CD4 expression in CD3^+^ CD4^+^ spleen cells (A–F). Numbers indicate percentages of CD4^+^ CD25^high^ CD3^+^ cells. Total cell counts (G) of CD4^+^ CD25^high^ cells in spleen from mice in (A–F) are shown as mean ± SD. The data are representative of three independent experiments.
(369 KB AI).

Figure S3 Effect of IL-4 on Already Existing Natural or Inducible T_reg_ Cells (A) CD4^+^CD25^high^ nT_reg_ cells were FACS-sorted and activated with plate-bound anti-CD3/CD28 plus IL-2 during 3 d and in the presence or absence of IL-4 (100 ng/ml) and harvested for real-time PCR analysis. The results shown represent the mean ± SD of three independent experiments.
(B) iT_reg_ cells were induced in vitro. FOXP3 espression was assessed by real-time PCR analysis in resting cells, in cells re-stimulated with plate-bound anti-CD3/CD28, with or without TGF-β, plus IL-2 during 3 d and in the presence (black bar) or absence (white bar) of IL-4 (100 ng/ml).
(C) Activation dramatically increases CD4^+^CD25^+^ T_reg_ cells suppressive capacity of CD4^+^CD25^+^ nT_reg_ cells. CD4^+^CD25^+^ nT_reg_ cells were preactivated during 2 d in the presence or absence of an increasing IL-4 concentration. After vigorous washing, their suppressive capacity on responder CD4^+^CD25^−^ was tested. IL-4 pretreatment did not affect the suppressive capacity of FACS-sorted CD4^+^CD25^high^ cells. 1 × 10^4^ CD4^+^CD25^+^ nT_reg_ cells were added to 5 × 10^4^ CD4^+^CD25^–^ and 5 × 10^4^ irradiated PBMCs. The results are representative of three independent experiments.
(269 KB AI).

Figure S4 Schematic Structure of the FOXP3 Gene and Location of the GATA3 Sites The scheme shows the location of the 11 exons spaced by a large intron (6000 bp) from the 5′untranslated region (UTR). Human, murine, and rat sequences are aligned and transcription start site (TSS) is indicated with an arrow.
(529 KB AI).

## Abbreviations

FACS: fluorescence activated cell sorting
IL-4: interleukin 4
iT_reg_: inducible T_reg_
mAb: monoclonal antibody
nT_reg_: natural, thymus-derived T_reg_
TCR: T cell receptor
TGF: transforming growth factor
T_reg_: T regulatory cell
rmIL-4: recombinant mouse IL-4
