# Supplementary figures and images for: GATA3-Driven Th2 Responses Inhibit TGF-β1–Induced FOXP3 Expression and the Formation of Regulatory T Cells
Source: PLoS Biol. 2007 Dec 27;5(12):e329. doi: 10.1371/journal.pbio.0050329 (PMC2222968; doi:10.1371/journal.pbio.0050329)

Figure S1

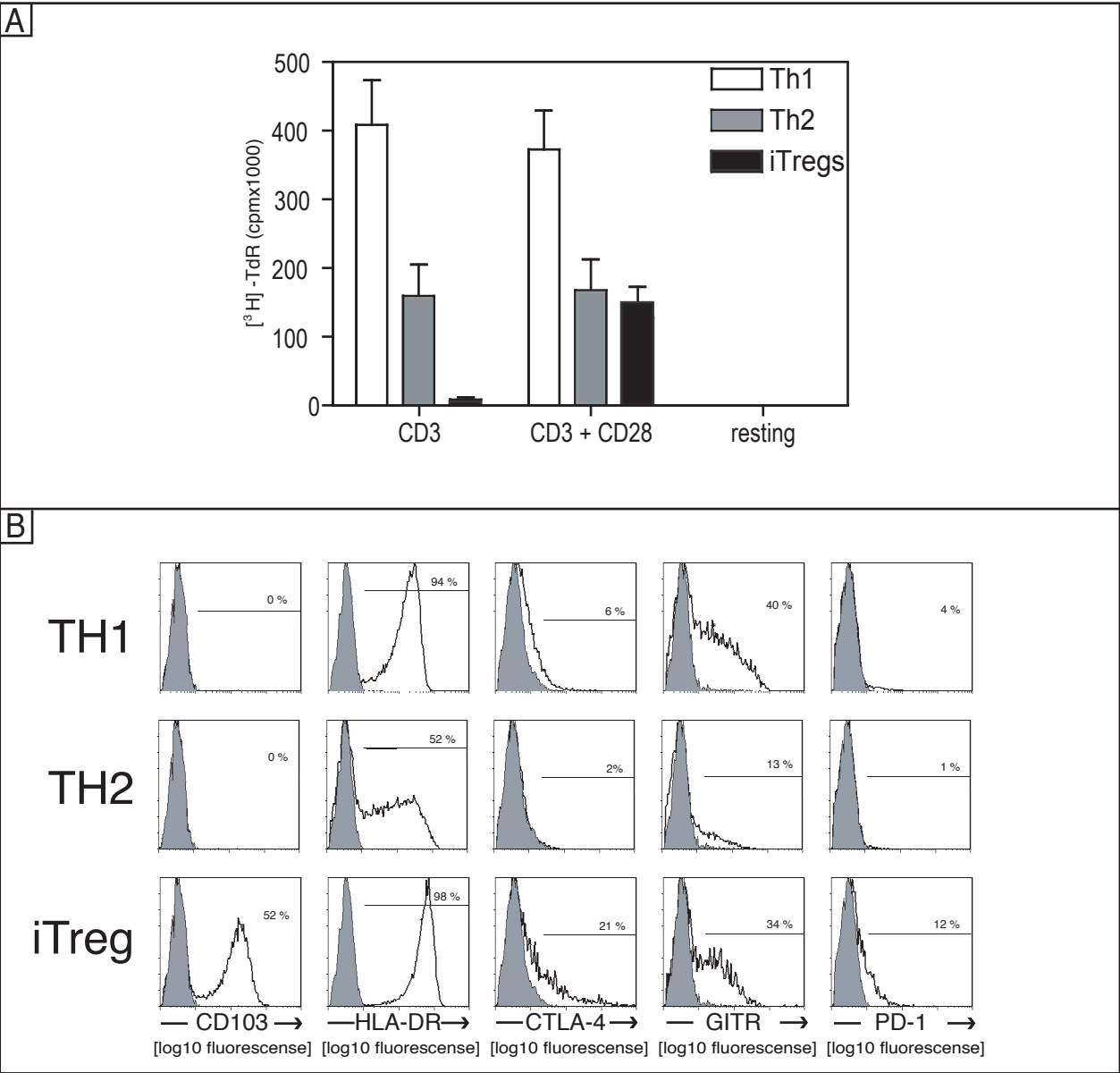

Supplement: Figure S1 — After two round of differentiation cultures, T cells were stimulated by plate-immobilized anti-CD3/CD28 and 3H-thymidine incorporation as measurement of proliferation was analyzed after 3 d of culture (A). In parallel, T cells were analyzed for Treg relevant surface receptor expression as indicated on the x-axis (B). (1.0 MB AI). [file pbio.0050329.sg001.pdf]

Figure S2

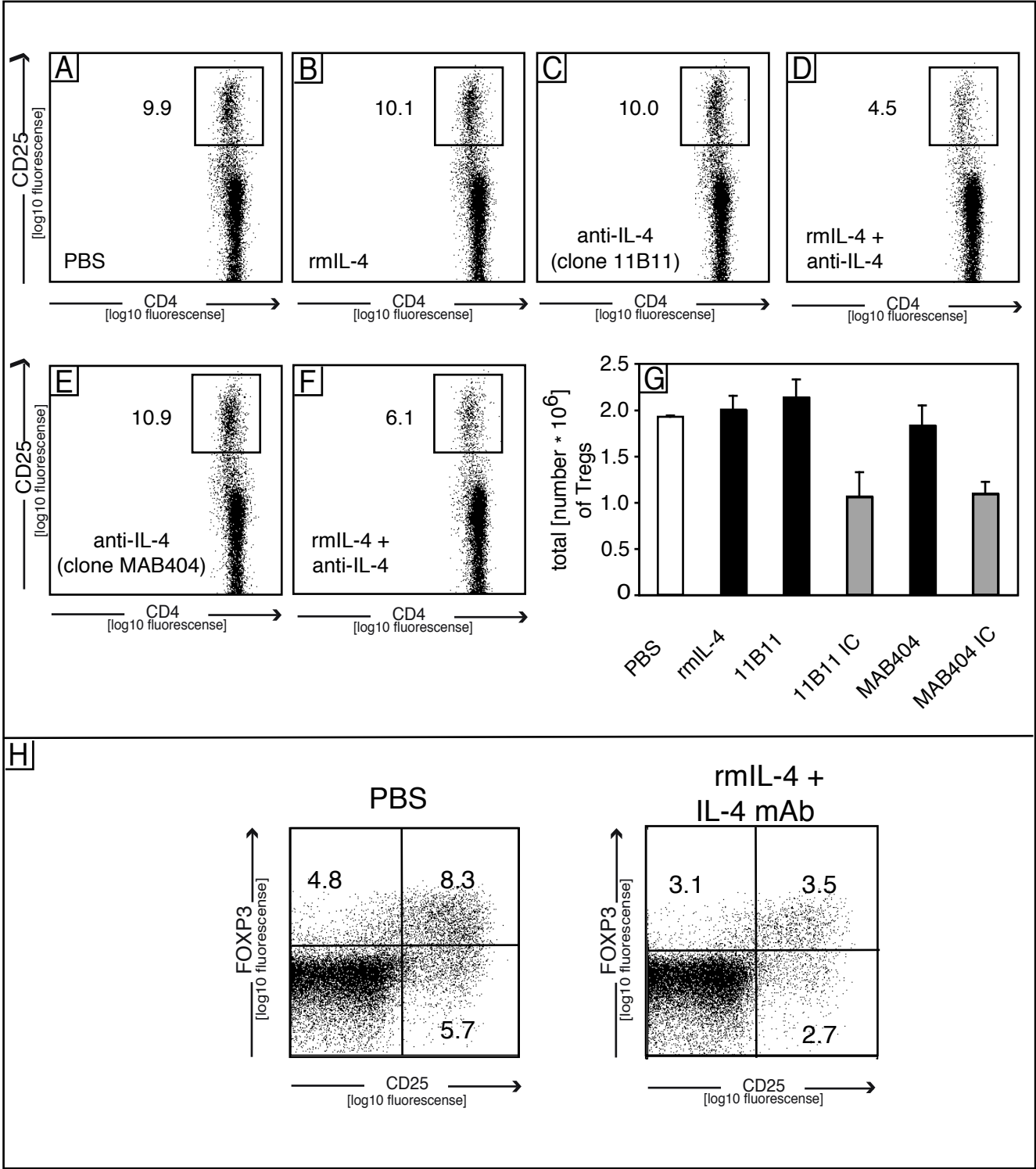

Supplement: Figure S2 — B6 mice were given every other day ip injections of phosphate-buffered saline (PBS), recombinant mouse IL-4 (rmIL-4), anti-IL-4 mAb (anti-IL-4 mAb, 11B11, or MAB404), or a mixture of rmIL-4 plus anti-IL-4 mAbs (11B11 or MAB404). Mice were analyzed on day 7 by flow cytometry for CD3, CD4, and CD25 expression. Shown is CD25 versus CD4 expression in CD3+ CD4+ spleen cells (A–F). Numbers indicate percentages of CD4+ CD25high CD3+ cells. Total cell counts (G) of CD4+ CD25high cells in spleen from mice in (A–F) are shown as mean ± SD. The data are representative of three independent experiments. (369 KB AI). [file pbio.0050329.sg002.pdf]

Figure S3

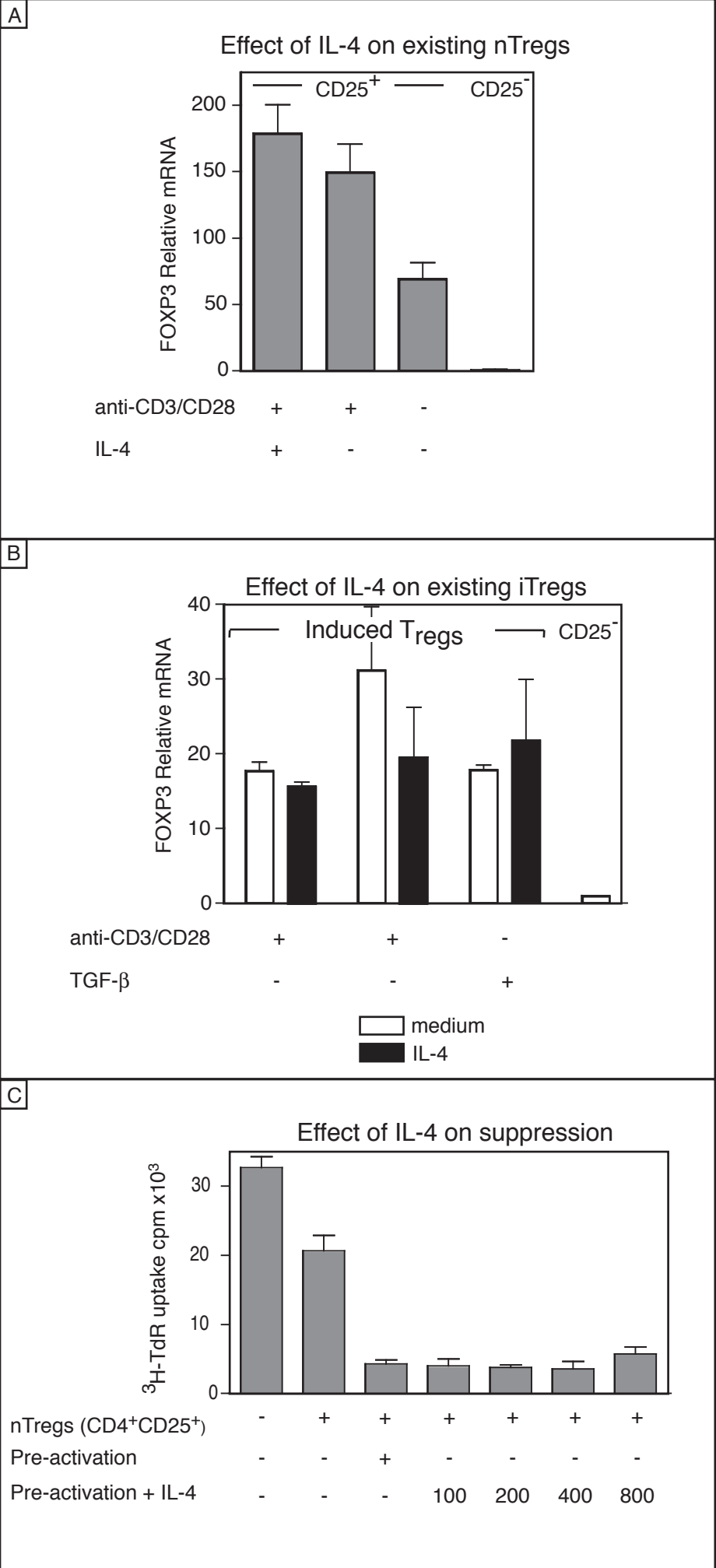

Supplement: Figure S3 — (A) CD4+CD25high nTreg cells were FACS-sorted and activated with plate-bound anti-CD3/CD28 plus IL-2 during 3 d and in the presence or absence of IL-4 (100 ng/ml) and harvested for real-time PCR analysis. The results shown represent the mean ± SD of three independent experiments. (B) iTreg cells were induced in vitro. FOXP3 espression was assessed by real-time PCR analysis in resting cells, in cells re-stimulated with plate-bound anti-CD3/CD28, with or without TGF-β, plus IL-2 during 3 d and in the presence (black bar) or absence (white bar) of IL-4 (100 ng/ml). (C) Activation dramatically increases CD4+CD25+ Treg cells suppressive capacity of CD4+CD25+ nTreg cells. CD4+CD25+ nTreg cells were preactivated during 2 d in the presence or absence of an increasing IL-4 concentration. After vigorous washing, their suppressive capacity on responder CD4+CD25− was tested. IL-4 pretreatment did not affect the suppressive capacity of FACS-sorted CD4+CD25high cells. 1 × 10^4^ CD4+CD25+ nTreg cells were added to 5 × 10^4^ CD4+CD25– and 5 × 10^4^ irradiated PBMCs. The results are representative of three independent experiments. (269 KB AI). [file pbio.0050329.sg003.pdf]

Figure S4

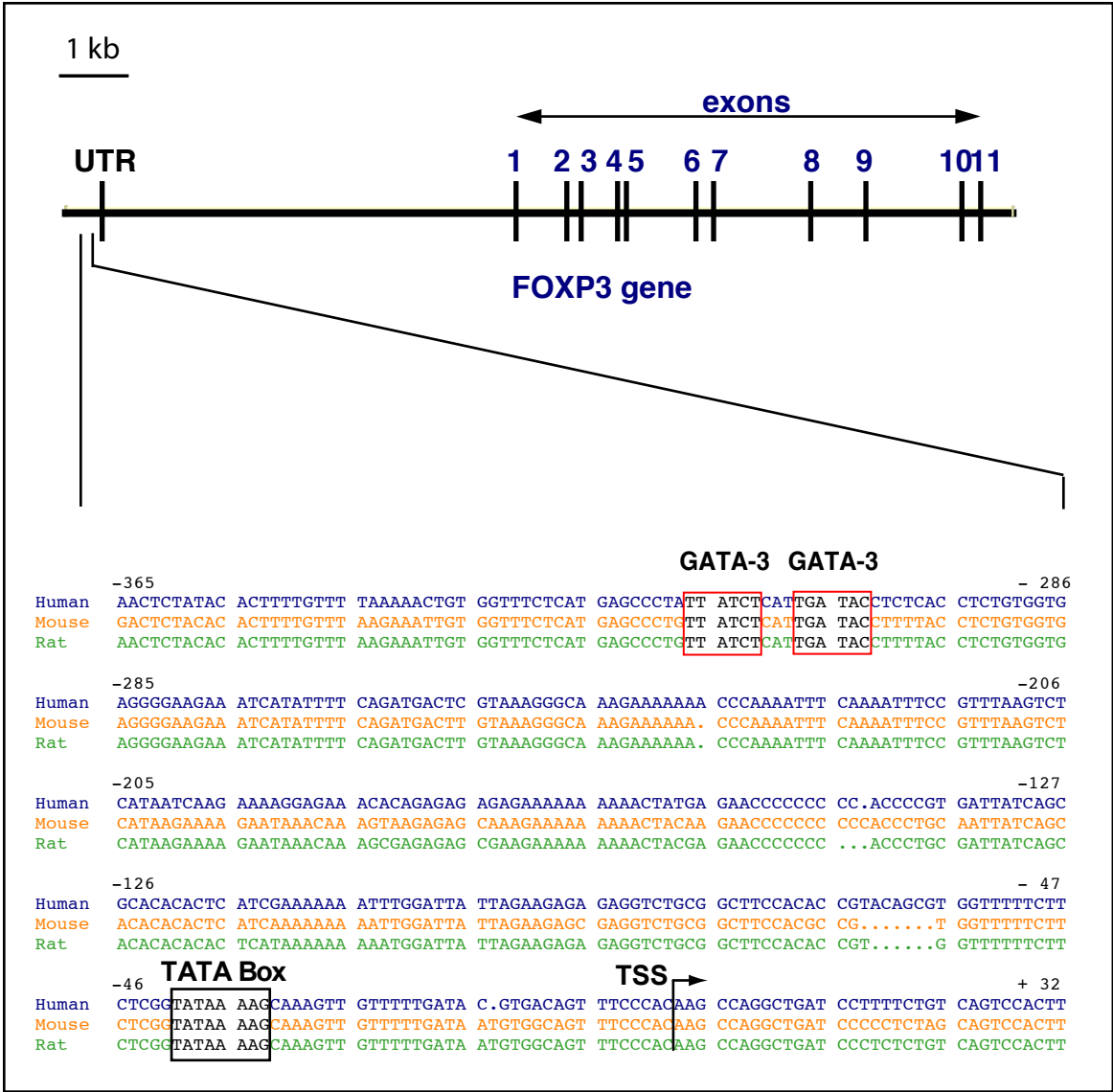

Supplement: Figure S4 — The scheme shows the location of the 11 exons spaced by a large intron (6000 bp) from the 5′untranslated region (UTR). Human, murine, and rat sequences are aligned and transcription start site (TSS) is indicated with an arrow. (529 KB AI). [file pbio.0050329.sg004.pdf]
